# Fibrillin-1 Regulates Arteriole Integrity in the Retina

**DOI:** 10.3390/biom12101330

**Published:** 2022-09-20

**Authors:** Florian Alonso, Ling Li, Isabelle Fremaux, Dieter Peter Reinhardt, Elisabeth Génot

**Affiliations:** 1 Centre de Recherche Cardio-Thoracique de Bordeaux, U1045, University of Bordeaux, INSERM, F-33000 Bordeaux, France; 2 Faculty of Medicine and Health Sciences, McGill University, Montreal, QC H3A 0C7, Canada; 3 Faculty of Dental Medicine and Oral Health Sciences, McGill University, Montreal, QC H3A 0C7, Canada

**Keywords:** arterioles, basement membrane, endothelial cells, fibrillin-1, Marfan syndrome, MAGP1, vascular leakage, ocular disease

## Abstract

Fibrillin-1 is an extracellular matrix protein that assembles into microfibrils that provide critical functions in large blood vessels and other tissues. Mutations in the fibrillin-1 gene are associated with cardiovascular, ocular, and skeletal abnormalities in Marfan syndrome. Fibrillin-1 is a component of the wall of large arteries but has been poorly described in other vessels. We examined the microvasculature in the retina using wild type mice and two models of Marfan syndrome, *Fbn1*^C1041G/+^ and *Fbn1*^mgR/mgR^. In the mouse retina, fibrillin-1 was detected around arterioles, in close contact with the basement membrane, where it colocalized with MAGP1. Both a mutation in fibrillin-1 or fibrillin-1 underexpression characteristically altered the microvasculature. In *Fbn1*^C1041G/+^ and *Fbn1*^mgR/mgR^ mice, arterioles were enlarged with reduced MAGP1 deposition and focal loss of smooth muscle cell coverage. Losartan, which prevents aortic enlargement in *Fbn1^C1041G/+^* mice, prevented smooth muscle cell loss and vessel leakiness when administrated in a preventive mode. Moreover, losartan also partially rescued the defects in a curative mode. Thus, fibrillin-1/MAGP1 performs essential functions in arteriolar integrity and mutant fibrillin-1-induced defects can be prevented or partially rescued pharmacologically. These new findings could have implications for people with Marfan syndrome.

## 1. Introduction

Vascular function is determined by the structural and functional properties of the wall and conditions that adversely affect this balance induce pathological vessel remodeling [1]. The extracellular matrix (ECM) is an essential determinant of vessel homeostasis and integrity, but the molecular players and mechanisms involved are not fully understood.

Fibrillin-1 is a large extracellular matrix protein that assembles into microfibrils that fulfil various structural and instructive roles [2]. Fibrillin-1 microfibrils serve as a principal scaffold for tropoelastin deposition required for elastic-fiber formation, which mediates the elastic recoil of tissues such as the aorta [3]. Microfibrils are also found in non-elastin containing tissues where they appear to function as stress-bearing structures such as the ciliary zonules [4]. Microfibrils have also been shown to be associated with the basement membrane (BM), inserted to anchor epithelial or endothelial cells (ECs) to elastic fibers via cell binding domains [1,5,6,7]. Fibrillin-1 has a characteristic domain organization, composed of arrays of calcium binding epidermal growth factor-like (cbEGF) domains interspersed with transforming growth factor beta (TGF-β)-binding protein-like (TB) domains [8]. Several of these domains are associated with accessory proteins that confer additional functionality to microfibrils [9]. A prominent role is the extracellular control of growth factor bioavailability through interaction with LTBPs (TGF-β binding proteins) and with several bone morphogenic proteins (BMPs) [10]. Fibrillin-1 interacts with other ECM components including microfibril-associated glycoproteins (MAGPs, MAGP1, and MAGP2), fibronectin, versican, perlecan, several fibulins, several ADAMTS, and ADAMTS-like proteins [9]. Consequently, mutations in fibrillin-1 can affect the structure, assembly, or stability of microfibrils, impact cell–matrix interactions as well as cytokine bioavailability or cell mechanosensing [11].

Mutations in the human fibrillin-1 gene (*FBN1*) cause Marfan syndrome (MFS; Online Mendelian Inheritance in Man [OMIM] #154700, prevalence 1/5000 to 1/10,000). This disorder is associated with cardiovascular, ocular, and skeletal abnormalities [12,13]. Loss of the integrity of the aortic wall results in aneurysms and dissections [14]. The consequences of fibrillin-1 mutations in smaller vessels, however, remain to be explored. Several mouse models of MFS have been established via gene targeting or missense mutations, and have been instrumental in advancing the pathophysiologic understanding of mild and severe forms of MFS. Germ line mutations in fibrillin-1 lead to progressive connective tissue destruction as a result of fibrillin-1 fragmentation and/or reduced fibrillin-1 microfibril formation. Heterozygous mice with a missense mutation in a central cbEGF domain (*Fbn1*^C1041G/+^) reproduce several aspects of human MFS and are generally considered a mild MFS model [15,16,17,18]. The mgR mouse model (*Fbn1*^mgR^.^mgR^) is characterized by a hypomorphic fibrillin-1 expression (20–25% of wild type levels) that leads to MFS-like manifestations with a more severe phenotype [19].

To study the role of fibrillin-1 in small vessels, we analyzed the mouse retina of the MFS mouse models, a well-defined and widely used model to study the developing and mature microvasculature [20]. Vascularization in the retina occurs postnatally and in an orderly and stereotypical manner. Guided by fibronectin and vascular endothelial growth factor-A (VEGF-A) from astrocytes, the vascular network extends two-dimensionally across the retinal surface between postnatal day (P) P0 and P8, and thereafter, secondary and tertiary plexi develop. The retina is also propitious for studying the mature vasculature. Vessel homeostasis relies on basement membrane (BM) integrity and proper vessel coverage by mural cells. Collagen type IV (Col IV) and laminin isoforms are the major structural component of all BMs [21], forming a supportive substratum for endothelial cell (EC) attachment and survival. In the retina, pericytes are embedded in the BM where they communicate with capillary ECs through direct contacts and paracrine signaling, a function that is performed by vascular smooth muscle cells (VSMC) in arterioles.

Therapeutic options to treat individuals with MFS include prophylactic surgical repair of the aortic root, β-blockers that decrease hemodynamic stress, and losartan, an angiotensin-II type-1 (AT1) receptor blocker (ARB) [22,23]. Losartan reduces aortic root dilation in MFS mouse models including *Fbn1*^C1041G/+^ mice [24,25]. However, randomized clinical trials with MFS patients produced more heterogeneous results [26]. Losartan inhibits AT1 receptor mediated angiotensin-II signaling and TGF-β hyperactivity, commonly observed in murine and human MFS [16,24]. The precise molecular mechanism of losartan action on aortic aneurysm progression in MFS is yet to be established. The effects of losartan on the microvasculature of MFS mice have not been investigated yet.

In the present study, we revealed the presence of perivascular fibrillin-1 in arterioles in the developing and mature microvasculature of the retina. Using the two murine MFS models carrying mutations in the FBN gene, we showed that fibrillin-1 is a critical player in retinal arteriole homeostasis. In fibrillin-1 mutant mice, MAGP1 deposition is altered at the arterioles, highlighting a role for this fibrillin-1 binding partner in microvascular integrity. We reveal that losartan treatment rescues major aspects of the microvascular retinal phenotype in MFS adult mice.

## 2. Materials and Methods

### 2.1. Animals

*Fbn1*^C1041G/+^ mice, kindly provided by Dr. Hal Dietz, have been previously described [17]. Heterozygous mice were crossed with C57BL/6 mice for more than 10 generations and maintained on this background to ensure that the colonies were genetically similar. *Fbn1*^mgR/mgR^ mice were obtained from Dr. Francesco Ramirez and were described previously [19,27]. For preventive treatment, starting at 4 weeks of age, WT and *Fbn1*^C1041G/+^ mice were given losartan ad libitum in the drinking water at a final concentration of 0.3 g/L (20–30 mg/kg/day), or left untreated. The losartan solution was changed once a week. After 3 months of treatment, mice were sacrificed (by 4 months old), and eyes were collected for analysis. For curative treatment, losartan administration was initiated at 3 months of age, and after 6 months of treatment (losartan or placebo), mice were sacrificed (by 9 months old) and analyzed as above. The numbers of analyzed mice are indicated in each experiment.

### 2.2. Whole Retina Immunohistochemistry

Postnatal day 6 (P6), 10 weeks, 4-months, 9 months, and 1-year old mice were sacrificed, enucleated, and the eyes were fixed in 4% paraformaldehyde for 2 h at 4 °C. Retinas were dissected, then incubated for 2 h at room temperature in blocking buffer (PBS, 2% BSA, 0.2% Triton X-100). After three 20 min washes in Pblec buffer (PBS supplemented with 1 mM MgCl_2_, 1 mM MnCl_2_, 1 mM CaCl_2_, and 1% Triton X-100), retinas were incubated overnight at 4 °C with fluorescein labeled isolectin B4 (IB4; Vector Laboratories, FL-1201, 1:25) and antibodies diluted in blocking buffer. Retinas were washed three times with blocking buffer and incubated with species-specific fluorescently labeled secondary antibodies (Jackson Laboratories, 1:100) diluted in blocking buffer for 2 h at room temperature. After three washes in PBS, whole retinas were flat-mounted in ProLong Gold Antifade reagent (Life Technologies) containing Hoechst 33,342 (Life Technologies) and analyzed with an epifluorescence microscope (Nikon TE-2000) or a laser scanning fluorescence microscope (Zeiss LSM 510 Meta inverted).

### 2.3. Antibodies for Immunolabeling

A polyclonal antiserum against mouse fibrillin-1 was produced in rabbit. Anti-fibrillin-1 antibodies were affinity-purified and antibodies cross-reacting with fibronectin were removed by absorption, resulting in monospecific anti-fibrillin-1 antibodies [4]. Antibodies against laminin (γ1-chain, clone 3E10, sc-65643) were obtained from Santa Cruz. Antibodies against MAGP2 (MBS2028486) were from Mybiosource. Antibodies against αSMA (clone 1A4, C6198) were purchased from Sigma. Col IV antibodies were obtained from BioRad (#2150-1470). Antibodies against VE-cadherin (clone 11D4.1, #555289) and CD31 (clone MEC13.3, #553370) were obtained from BD Pharmingen. Antibodies against MAGP1 (AF4977) and albumin (AF3329) were purchased from R&D Systems. Tropoelastin antibodies were a gift from Dr. Robert P. Mecham. For secondary detection, species-specific fluorescently labeled secondary antibodies (Jackson ImmunoResearch) were used (see Supplementary Table S1 for a list of the antibodies used in the study).

### 2.4. Analysis of the Retinal Vasculature

To quantify the diameters of retinal arterioles and venules, collateral angles, arteriolar EC areas and Col IV, Laminin, αSMA, plasmatic albumin, VE-Cadherin, fibrillin-1, MAGP1, MAGP2 and elastin, a z-stack confocal series of 30 optical sections (x/y/z = 146.25 × 146.25 × 0.3 μm) were acquired with a 63× oil immersion objective. Acquisitions were performed at the area of the first collateral vessel bifurcation starting from the optic disc. The maximal intensity projections of the confocal stacks were analyzed using ImageJ software [28]. The diameters of retinal arterioles and venules were calculated from three different length measurements performed perpendicularly to the vessel axis at the first collateral vessel bifurcation. The collateral angles of retinal arterioles were determined (at the first collateral vessel bifurcation) using the ImageJ “Angle tool” function. The positive surface areas per field were measured on thresholded images and normalized to the IB4-positive surface area. For 3D reconstructions, confocal stacks were first deconvoluted using the AutoQuant X3 software (Media Cybernetics) and then processed for image reconstruction using IMARIS software (Bitplane).

### 2.5. Statistics

Statistical analysis was performed with GraphPad Prism 6 (GraphPad Software, Inc., San Diego, CA, USA). Data represent at least three independent experiments. Graphs are presented as mean values ± SD (bars) and individual values (data points in scatter plot with bar). Significance was determined with the Student’s t-test or the one-way ANOVA, followed by Tukey’s post-tests. *p* values < 0.05 were considered statistically significant.

## 3. Results

### 3.1. Fibrillin-1 Is Detected in the Arteriolar Wall and Its Deficiency Affects the Integrity of Arterioles

**Figure 1:**
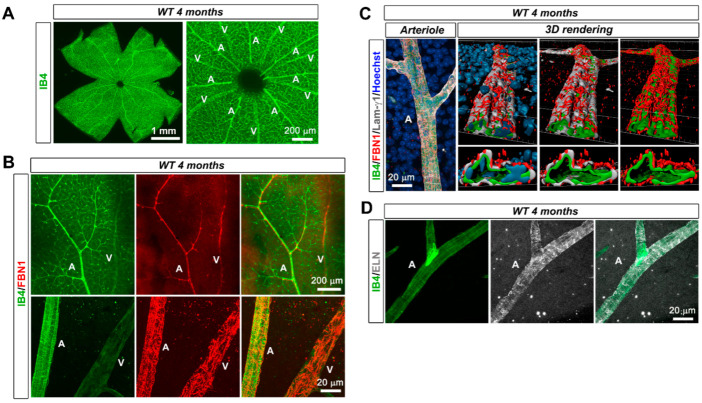
Fibrillin-1 is expressed within the microvasculature of the adult mouse retina. (**A**) IB4 (green) stained retinal whole-mounts from a 4 month old WT mouse (**left panel**). Higher magnification image at the optic disc area (**right panel**) shows retinal capillaries of the primary vascular plexus and the positioning of arterioles and venules in alternating pattern. (**B**) FBN1 (red) and IB4 (green) staining in a single petal of a flat mounted retina from a 4 month old WT mouse (**top panels**). Higher magnification images at the optic disc area reveal FBN1 around large arterioles and venules (**bottom panels**), (*n* = 9 WT mice). (**C**) High magnification image of an arteriole from WT retinal whole mounts at 4-month labeled for FBN1 (red), IB4 (green), laminin-γ1 (white), and nucleus (Hoechst, blue) (**left panel**). The FBN1 network is closely associated with the endothelial BM (**right panels**) as observed after 3D volume reconstruction of the same area, (*n* = 9 WT mice). (**D**) IB4 (green) and ELN (white)-stained retinal vessels from a 4 month old WT mouse. A—arteriole and V— venule.

The retinal microvasculature of the primary plexus from the adult wild type mouse (4-month-old animals) was analyzed by immunofluorescent staining of flat-mounted retinas. In endothelial marker isolectin B4 (IB4)-stained retinas, fibrillin-1 was observed around arterioles and veins but not around capillaries (Figure 1A,B). The protein was detected as a mesh around arterioles, closely associated with the BM (Figure 1C and Movie S1). Elastin staining correlated with the arteriole-associated distribution of fibrillin-1 (Figure 1D). In age-matched *Fbn1*^C1041G/+^ mice, fibrillin-1 and elastin immunostaining at the arterioles was reduced by ~40–65% compared to the wild type mice (Figure S1A,B).

**Figure 2:**
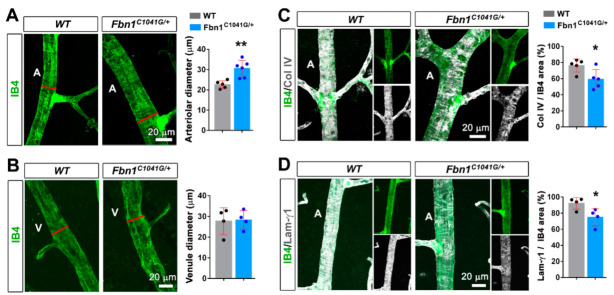
The fibrillin-1 C1041G mutation impairs retinal arteriole integrity in adult mice. (**A**) IB4 (green) stained arteriole from *Fbn1^C1041G/+^* retinas at 4 months (**right panel**) vs. WT (**left panel**). Quantification of the arteriolar diameter, measured at the first bifurcation (red line), (*n* = 5 WT and 6 *Fbn1^C1041G/+^* mice). (**B**) Same experimental setup as in (**A**) for the venule diameter, (*n* = 4 WT and 4 *Fbn1^C1041G/+^* mice). (**C**) IB4 (green) and Col IV (white) stained arteriole from Fbn1^C1041G/+^ retinas at 4 months (**right panels**) vs. WT (**left panels**). Quantification of the Col IV positive area over the IB4 positive area is shown (*n* = 5 WT and 5 *Fbn1^C1041G/+^* mice). (**D**) The same experimental setup as in (**C**) for IB4 (green) and laminin-γ1 (white), (*n* = 4 WT and 4 *Fbn1^C1041G/+^* mice). A—arteriole and V—venule. * *p* < 0.05; ** *p* < 0.01 vs. WT mice (Student’s *t* tests).

Strikingly, we observed an enlargement of the arteriolar diameter in mutant mice vs. wild type (WT), and this was not observed in veins (Figure 2A,B). The bifurcation angle between the arteriole and daughter vessels (measured at the first bifurcation) tended to be larger in the *Fbn1*^C1041G/+^ mice, consistent with the arteriolar widening, although the analysis did not reach significance (Figure S1C). Col IV and laminin (γ1 chain staining) were reduced in comparison with the WT mice (Figure 2C,D), underpinning altered BM integrity.

**Figure 3:**
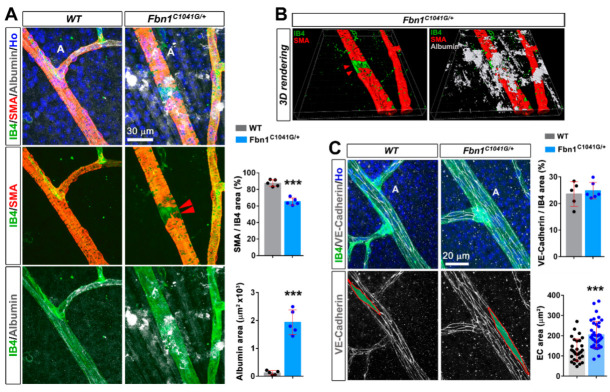
The fibrillin-1 C1041G mutation impairs retinal arteriole integrity and functionality in adult mice. (**A**) Retinal arteriole from 4 month old WT (**left panels**) and *Fbn1^C1041G/+^* (**right panels**) mice stained for the VSMC marker αSMA (red), IB4 (green), albumin (white) and the nucleus marker Hoechst (blue). Note the focal loss of VSMCs around the retinal arteriole (**middle panels**, red arrowheads) and extravasated albumin (**bottom panels**) in 4 month old *Fbn1^C1041G/+^* mice. Quantifications of the αSMA positive area over the IB4 positive area and albumin area per field are shown, (*n* = 5 WT and 5 *Fbn1^C1041G/+^* mice). (**B**) 3D volume reconstruction of the same image of the *Fbn1^C1041G/+^* arteriole displayed in (**A**) showing focal loss of VSMCs (**left panel**, red arrowheads) and extravasated albumin (**right panel**). (**C**) Retina whole-mounts from 4 month old WT (**left panels**) and *Fbn1^C1041G/+^* mice (**right panels**) stained for IB4 (green), the adherens junction marker VE-cadherin (white) and the nucleus marker Hoechst (Ho, blue) (**top pane**l). The single VE-cadherin channel is shown (**bottom panels**). The red line delineates the contours of a single endothelial cell. Quantifications of the VE-cadherin positive area over the IB4 positive area and the mean EC surface area are shown (*n* = 30 ECs from 5 WT and 39 ECs from 6 *Fbn1^C1041G/+^* mice). A—arteriole. *** *p* < 0.001 vs. WT mice (Student’s *t* tests).

To investigate the causes of arteriole enlargement, we examined whether the C1041G mutant fibrillin-1 affected the contractile smooth vascular cell (VSMC) coverage. Retinal arterioles displayed uniform immunoreactivity for the VSMC marker αSMA, and for the EC marker IB4, in WT retinas (Figure 3A). In the *Fbn1*^C1041G/+^ mice, while IB4 staining remained uniform, focal loss of αSMA coverage around the retinal arterioles was apparent and widening of the arterial diameter was exacerbated in these regions (Figure S1D and Figure 3A,B). Diffuse albumin staining along arterioles and more particularly in regions corresponding to poor VSMC coverage indicated plasma protein extravasation from leaky vessels (Figure 3A,B and Movie S2) in the retinas from *Fbn1*^C1041G/+^ mice. Albumin was not detected around vessels in the control WT retinas (Figure 3A). In regions devoid of αSMA coverage, VE-cadherin staining did not reveal any gross defect of cell–cell junctions, but EC areas were larger than in the control vessels (Figure 3C). Collectively, these results show that altered BM integrity and/or focal or segmental loss of VSMCs are involved in the vessel permeability defect observed in the retinas from *Fbn1*^C1041G/+^ mice. We did not detect arteriovenous malformation associated with arteriole enlargement.

**Figure 4:**
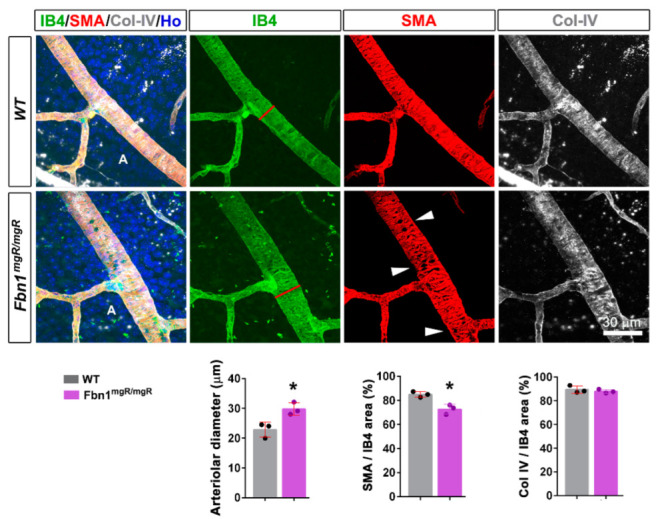
Underexpression of fibrillin-1 similarly leads to alterations in the arteriole diameter and αSMA coverage in *Fbn1^mgR/mgR^* mice. Retinal arteriole from 10 week old WT (**top panels**) and *Fbn1^mgR/mgR^* (**bottom panels**) mice stained for the VSMC marker αSMA (red), IB4 (green), Col IV (white), and the nucleus marker Hoechst (blue). Note the increased arteriolar diameter (red line) and the disorganized VSMCs around the retinal arteriole (white arrowheads). Quantifications of the arteriolar diameter and of the αSMA and Col IV positive areas over the IB4 positive area are shown (*n* = 3 WT and 3 *Fbn1^mgR/mgR^* mice). A—arteriole. * *p* < 0.05 vs. WT mice (Student’s *t* tests).

To determine whether the defects resulted from the C1041G mutation or from altered fibrillin-1 deposition, similar experiments were carried out in the *Fbn1*^mgR/mgR^ model. Figure 4 shows that underexpression of fibrillin-1 similarly leads to alterations in arteriole diameters and αSMA coverage in this MFS model, indicating that the defects were associated with reduced perivascular deposition of fibrillin-1. Interestingly, Col IV staining was not significantly affected in the *Fbn1*^mgR/mgR^ model at this stage.

### 3.2. Fibrillin-1 Deficiency Also Affects the Integrity during Development and Aging of the Retinal Vasculature

**Figure 5:**
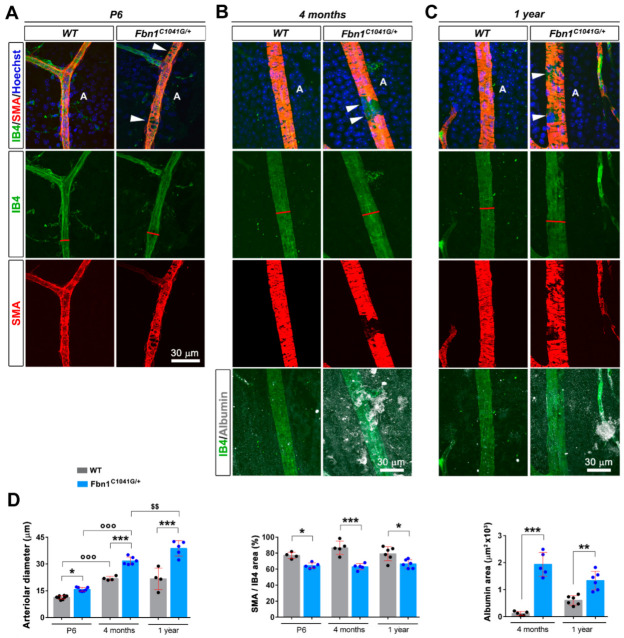
The fibrillin-1 C1041G mutation progressively impairs retinal arteriole integrity and functionality, compromising VSMC coverage and endothelial barrier function. (**A**) IB4 (green), αSMA (red), and Hoechst (blue) stained arteriole from P6 *Fbn1^C1041G/+^* retinas (**right panels**) compared to WT (**left panels**). (**B**,**C**) IB4 (green), αSMA (red), albumin (white), and Hoechst (blue) stained arteriole from 4-month- (**B**) and 1-year-old (**C**) WT (**left panels**) and *Fbn1^C1041G/+^* retinas (**right panels**). (**D**) Quantitative assessment of the arteriolar diameter (**left panel**), αSMA positive area over the IB4 positive area (**middle panel**), and albumin positive area per field (**right panel**) according to age in the WT and *Fbn1^C1041G/+^* mice. White arrowheads—focal loss of VSMCs. A—arteriole. * *p* < 0.05; ** *p* < 0.01; *** *p* < 0.001 vs. respective WT mice. °°° *p* < 0.001 vs. respective P6 mice. $$ *p* < 0.01 vs. respective 4-month-old mice (one-way ANOVA followed by Tukey’s post-test).

We then performed the same analysis with animals aged 6 days (P6) or one year to determine whether these defects were early and if they evolved with age (Figure 5). The analysis showed that the defects in arteriole diameters and αSMA coverage were already present at P6, and that arteriole diameter enlargement is aggravated with age (Figure 5A–D). In IB4-stained retinas at P6, fibrillin-1 was easily detected around arterioles (Figure 5A,B), but in contrast with the adult vasculature, was not detected around veins at this stage (Movie S3, Figure S2). Albumin leakage tended to be more pronounced in 1-year- vs. 4-month-old WT animals (not significant), although the defect was milder in the vasculature from 1-year-old mutant mice vs. 4 month-old mice (significant but not highlighted in the figure) (Figure 5D). Increased arteriolar diameter and defective VSMC coverage was also observed in *Fbn1*^mgR/mgR^ mice at p6 (Figure S3). Microfibril-associated glycoproteins, MAGP1 and MAGP2, are small ECM proteins that associate with fibrillins to influence microfibril function. Interestingly, simultaneous inactivation of their genes (MAGP1/2 double knockout mice) show age-dependent aortic dilation [29]. In the adult, MAGP1 has a wider tissue distribution than MAGP2. However, both genes are known to be equally expressed at p6 in the aorta [30] and we show here that both proteins were detected in the retinal arterioles at this stage (Figure S4A,B). MAGP2 staining was evenly detected in the vasculature (Figure S4A). In contrast, MAGP1 was more closely associated in the fibrillin-1 enriched arterioles. Whereas MAGP2 immunostaining did not detect significant difference between *Fbn1*^C1041G/+^ and WT retinal vasculature (Figure S4A), MAGP1 immunostaining was weaker in both *Fbn1*^C1041G/+^ and *Fbn1*^mgR/mgR^ retinas and its pattern matched that of fibrillin-1 (Figure S4B,C).

### 3.3. Focal Loss of VSMC Coverage, Poor BM Protein Coverage, and Vessel Leakiness Are Prevented by Losartan Treatment

**Figure 6:**
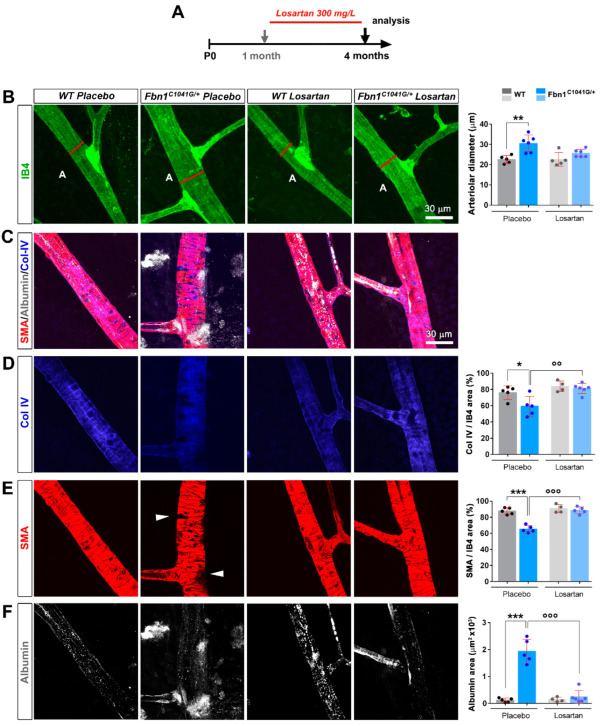
Early losartan treatment prevents the structural and functional defects in the retinal arteriole of adult *Fbn1^C1041G/+^* mice. (**A**) Schematic representation of the protocol of preventive losartan treatment in WT and *Fbn1^C1041G/+^* animals. The treatment was initiated at 1 month for a duration of 3 months. (**B**) IB4 (green) stained retinal arteriole from 4-month-old WT and *Fbn1^C1041G/+^* mice treated or not with losartan. Quantification of the arteriolar diameter, measured at the first bifurcation (red line) is shown, (*n* = 5 H_2_O-treated WT; 5 H_2_O-treated *Fbn1^C1041G/+^*; 5 losartan-treated WT; 6 losartan-treated *Fbn1^C1041G/+^* mice). (**C**) αSMA (red), albumin (white), and Col IV (blue) stained retinal arteriole from 4-month-old WT and *Fbn1^C1041G/+^* mice treated or not with losartan. (**D**) Images of the single Col IV channel (blue) from the merged pictures shown in (**C**). Quantification of the Col IV positive area over the IB4 positive area is shown, (*n* = 5 H_2_O-treated WT; 5 H_2_O-treated *Fbn1^C1041G/+^*; 4 losartan-treated WT; 6 losartan-treated *Fbn1^C1041G/+^* mice). (**E**,**F**) The same experimental setup as in (**D**) for αSMA (red) in (**E**) and for albumin (white) in (**F**). White arrowheads—focal loss of VSMCs, A—arteriole. * *p* < 0.05; ** *p* < 0.01; *** *p* < 0.001 vs. respective WT mice. °° *p* < 0.01; °°° *p* < 0.001 vs. respective untreated mice (one-way ANOVA followed by Tukey’s post-test).

Losartan was shown to prevent aortic enlargement in the *Fbn1*^C1041G/+^ mouse model [16]. Whether losartan affects the microvasculature of these mice has not been investigated yet. We therefore examined whether the structural and functional vascular defects detected in the retinas from *Fbn1*^C1041G/+^ mice were ameliorated by losartan treatment. The drug crossed the blood–retinal barrier and was shown to be delivered to the eye [31]. Losartan treatment was initiated in one-month old animals, continued for 3 months, then the mice were sacrificed for retinal vasculature analysis (Figure 6A).

IB4 staining revealed reduced arteriole diameter, coincident loss of diffuse albumin staining, and restoration of both BM Col-IV/laminin and αSMA coverages in the losartan-treated *Fbn1*^C1041G/+^ mice relative to the placebo group (Figure 6B–F). Interestingly, losartan treatment improved MAGP1 deposition/coverage of arterioles in the retinas of *Fbn1*^C1041G/+^ mice despite the alteration of the fibrillin-1 and elastin status in the MFS model, which persisted upon losartan treatment (Figure S5A–C). Altogether, the results show that losartan treatment has significant beneficial effects on mutated fibrillin-1-induced retinal arteriolar defects in this MFS model.

### 3.4. Focal Loss of VSMC Coverage, Poor BM Protein Coverage, and Vessel Leakiness Are Reverted by Losartan Treatment

**Figure 7:**
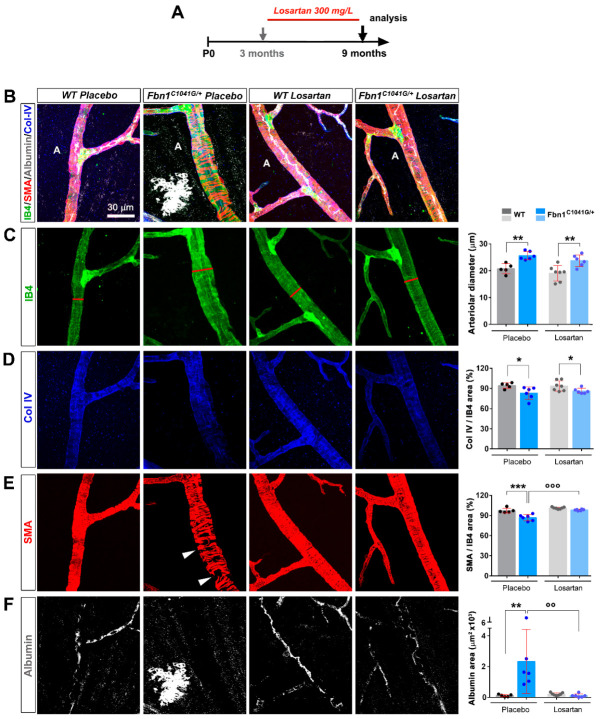
Late losartan treatment rescues the functional defects in the retinal arteriole of adult *Fbn1^C1041G/+^* mice. (**A**) Schematic representation of the protocol of preventive losartan treatment in WT and *Fbn1^C1041G/+^* animals. The treatment was initiated at 3 months for a duration of 6 months. (**B**) IB4 (green), αSMA (red), serum albumin (white), and Col IV (blue) stained retinal arteriole from 9-month-old WT and *Fbn1^C1041G/+^* mice treated or not with losartan. (**C**) Images of the single IB4 channel (green) from the merged pictures shown in (**B**). Quantification of the arteriolar diameter, measured at the first bifurcation (red line) is shown (*n* = 5 H_2_O-treated WT; 6 H_2_O-treated *Fbn1^C1041G/+^*; 7 losartan-treated WT; 6 losartan-treated *Fbn1^C1041G/+^* mice). **D.** Images of the single Col IV channel (blue) from the merged pictures shown in (**B**). Quantification of the Col IV positive area over the IB4 positive area is shown.). (**E**,**F**) Same experimental setup as in (**D**) for αSMA (red) in (**E**) and for albumin (white) in (**F**). White arrowheads—focal loss of VSMCs. A—arteriole. * *p* < 0.05; ** *p* < 0.01; *** *p* < 0.001 vs. respective WT mice. °° *p* < 0.01; °°° *p* < 0.001 vs. respective untreated mice (one-way ANOVA followed by Tukey’s post-test).

We next examined whether the structural and functional arteriolar defects detected in the retinas of *Fbn1*^C1041G/+^ mice could be reversed by losartan treatment. Losartan treatment was initiated in 3-month-old animals in which vascular defects had developed, administrated over a 6-month period, and then the mice were sacrificed for retinal vasculature analysis (Figure 7A). IB4 staining revealed no difference in the arteriole diameter or BM-Col IV coverage but a significant decrease in diffuse albumin staining around arterioles and restoration of αSMA coverages in losartan-treated *Fbn1*^C1041G/+^ mice relative to the placebo group (Figure 7B–F). Altogether, the results show that losartan treatment has significant beneficial effects on mutated fibrillin-1-induced arteriolar defects in the retinas. However, losartan is effective on more arteriolar defects when administered in a preventive mode compared to a curative mode.

## 4. Discussion

### 4.1. The Retinal Arterioles Are Sheathed by Fibrillin-1 and MAGP1 during Their Formation and at Maturity

Microfibrils are components of large arteries supporting elastic fiber formation and in turn elastic recoil. We herein describe the localization of fibrillin-1 around the retinal arterioles. Fibrillin-1 was also detected around veins in adult mice, but not during development (at P6). The microfibril-associated proteins MAGP1 and MAGP2, which directly interact with fibrillin-1 [32,33,34], were found to colocalize with fibrillin-1 at the retinal arterioles. The MAGP2 expression pattern was not altered in the vasculature of the *Fbn1*^C1041G/+^ retinas. In contrast, MAGP1 and fibrillin-1 staining more closely overlapped. The distinctive pattern of MAGP1 and MAGP2 localization supports the distinct functions of the two proteins in arteriolar homeostasis. One MAGP1 binding site localizes close to the *n*-terminus of fibrillin-1 [32], and a second one more centrally in the region of TB3-cbEGF11 [34].

### 4.2. Mutant Fibrillin-1 Alters Arteriolar Integrity

Fibrillin-1 is detected as early as P6 and is maintained in the adult vasculature. We detected a number of abnormalities in arterioles from MFS mice including arteriole enlargement, focal loss of VSMCs, and increased permeability in *Fbn1*^C1041G/+^ mice. This was associated with a reduced deposition of MAGP1 in the *Fbn1*^C1041G/+^ mice. Interestingly, in mutant mice, arteriole enlargement is not a defect associated with aging, but already exists at the onset of vascularization. In the *Fbn1*^C1041G/+^ adult mice, we showed that the defects in VSMC coverage were associated with increased vascular permeability. Reduced Col IV coverage was observed in the *Fbn1*^C1041G/+^ mice but not in the *Fbn1*^mgR/mgR^ model, suggesting that reduced VSMC coverage is a more prominent and early consequence of fibrillin-1 deficiency and does not result from the BM Col IV defect. The arteriolar defects observed in the mouse models may underly some of the retinal lesions observed in individuals with MFS [35,36,37]. Marfan patients may have discrete vascular abnormalities promoting focal lesions that have thus far not been recognized. Arteriole enlargement is reminiscent of the aortic defect in MFS disease (i.e., ascending aortic wall dilation and aneurysm formation). Aortic aneurysms in MFS are characteristically associated with fragmentation of the elastic lamellae [18]. In adult mice, fibrillin-1 was also detected at venules, but dilation was not observed in these vessels. As the blood pressure is much higher in arterioles than in venules, arteriole widening may be associated with a defective vasomotor defect as reported for small arteries and impaired endothelial function [38,39].

### 4.3. Losartan Rescues Mutant Fibrillin-1 Induced Defects

We found that losartan prevented the majority of arteriole defects in the *Fbn1*^C1041G/+^ MFS mice including dilation, leakiness, BM, and VSMC coverage when administered at an early stage of the disease. Losartan did not reduce the increased arteriolar bifurcation angles, suggesting that the beneficial effect was not mediated by its action on blood pressure or endothelial dysfunction [40]. Once the vascular defects were established, losartan had therapeutically beneficial effects, being able to restore VSMC coverage and reduce the vascular permeability defect. Interestingly, MAGP1 staining improved in losartan-treated animals, suggesting that impaired MAGP1 deposition may underly the observed vascular alterations, and that losartan somehow increased the production and/or deposition of the protein despite the altered fibrillin-1 status. MAGP1 may therefore enhance the performance of mutant-fibrillin-1 containing microfibrils. Of note, previous studies have reported the temporal expression profile of MAGP1 and MAGP2 in mouse aorta or lung, demonstrating that MAGP1 exhibits an expression pattern similar to that of fibrillin-2, whereas MAGP2 follows the pattern of fibrillin-1 [30]. Interestingly, losartan treatment was shown to rescue vascular defects in MAGP1 deficient mice [41], to activate eNOS [42], and to increase nitric oxide production by ECs [40].

To which extent could these defects be similar to those found in the aorta? It is conceivable that plasma leakage also affects the integrity of larger arteries. Such abnormalities do not lead to detectable manifestations in these vessels, which are better protected by perivascular elements than arterioles. However, in such a scenario, losartan may also contribute to prevent large vessel deterioration and account for some of the beneficial effects reported for losartan treatment [24,25]. We provide here the first description of extended micro-arterial defects using the *Fbn1*^C1041G/+^ and *Fbn1*^mgR/mgR^ MFS mice that are characterized by progressive aortic aneurysms. In adult *Fbn1*^C1041G/+^ mice, most microvascular defects in the retina could be prevented with losartan treatment. Furthermore, losartan was able to restore to some extent the VSMC coverage and correct the vascular permeability defect when administrated in a curative mode. The rescue is associated with a significant increase in MAGP1 deposition in the altered fibrillin-1 matrix. In zebrafish, morpholino inactivation of MAGP1 leads to dilated vessels in the eyes such as morpholino inactivation of fibrillin-1, which supports the hypothesis that the two proteins operate in the same pathway [43]. Like fibrillins, MAGPs also control TGF-β signaling [44,45]. Whereas fibrillin-1 covalently binds inactive latent TGF-β through several LTBPs, MAGP1 non-covalently binds active TGF-β [46]. MAGP1 could serve to dampen TGF-β signaling by trapping active TGF-β from the immediate membrane microenvironment. In MFS mice, a reduction in MAGP1 deposition would therefore increase the TGF-β activity and disturb vascular homeostasis.

Losartan has been shown to overcome the mechanical deterioration of the elastin-rich aorta and lung tissues [47]. In addition, from studies in tumor models, it is known that losartan per se does not reduce vessel leakiness of abnormal vessel but acts on the vessel microenvironment [48]. Losartan may therefore alter the properties of the ECM so that MAGP1 is more retained in the modified matrix in MFS. Losartan may therefore mitigate TGF-β signaling both by blocking the angiotensin-II type 1 (AT1) receptor within the renin–angiotensin–aldosterone system and by a still unknown mechanism, restoring some MAGP1 neutralizing action on active TGF-β.

## 5. Conclusions

We provide evidence that fibrillin-1 mutation or underexpression progressively compromises the integrity of the retinal arteriole in mice. Losartan treatment is effective in preventing the arteriolar defects. Altogether, our data establish the instructive role of the microfibril proteins fibrillin-1 and MAGP1 in vessel homeostasis.

## Data Availability

All datasets of this study will be made available upon request to the corresponding authors.

## Supplementary Materials

The following supporting information can be downloaded at: https://www.mdpi.com/article/10.3390/biom12101330/s1, Figure S1: Expression of fibrillin-1 and deposition of elastin within the retinal arteriole of adult WT and *Fbn1*^C1041G/+^ mice; Figure S2: FBN1 expression within the microvasculature of the developing mouse retina; Figure S3: The underexpression of fibrillin-1 leads to early alterations in the arteriole diameter and αSMA coverage in the developing retina from P6 *Fbn1^mgR/mgR^* mice; Figure S4: Reduced FBN1 and MAGP1 but not MAGP2 expression/deposition in the developing retina from P6 *Fbn1*^C1041G/+^ and *Fbn1^mgR/mg^*^R^ mice; Figure S5: Expression/deposition of fibrillin-1, elastin and MAGP1 within the retinal arteriole of adult WT and *Fbn1*^C1041G/+^ mice treated or not with losartan; Video S1: The 3D volume reconstruction of the area shown in Figure 1C showing that FBN1 is detected as a network around arterioles in close interaction with the endothelial BM in retinas from 4-month-old wild type animals; Video S2: The 3D volume reconstruction of the same image of the *Fbn1*^C1041G/+^ arteriole shown in Figure 3B showing focal loss of VSMCs around the retinal arteriole and extravasated albumin in 4-month old *Fbn1*^C1041G/+^ mice; Video S3: The 3D reconstruction showing that FBN1 is detected around arterioles in the wild type retina at P6; Table S1: List of antibodies used in the study.
