# Supplementary material for: Fibrillin-1 Regulates Arteriole Integrity in the Retina
Source: Biomolecules. 2022 Sep 20;12(10):1330. doi: 10.3390/biom12101330 (PMC9599515; doi:10.3390/biom12101330)
Supplement: Supplementary file 1 [file biomolecules-12-01330-s001.zip › Supplementary materials-edited-1-11.pdf]

## Supplementary Materials

# Fibrillin-1 regulates arteriole integrity in the retina

**Florian Alonso [1]*, Ling Li [2], Isabelle Fremaux [1], Dieter P. Reinhardt [2,3] and Elisabeth Génot [1,*]**

[1] Univ. Bordeaux, INSERM, Centre de Recherche cardio-thoracique de Bordeaux, U1045, F-33000 Bordeaux. France

[2] Faculty of Medicine and Health Sciences and [3] Faculty of Dental Medicine and Oral Health Sciences, McGill University, Montreal, QC, Canada H3A 0C7

* Correspondence: elisabeth.genot@inserm.fr (E.G.); florian.alonso@u-bordeaux.fr (F.A.)

- **Figures S1-5**
- **Videos S1-3**
- **Table S1**

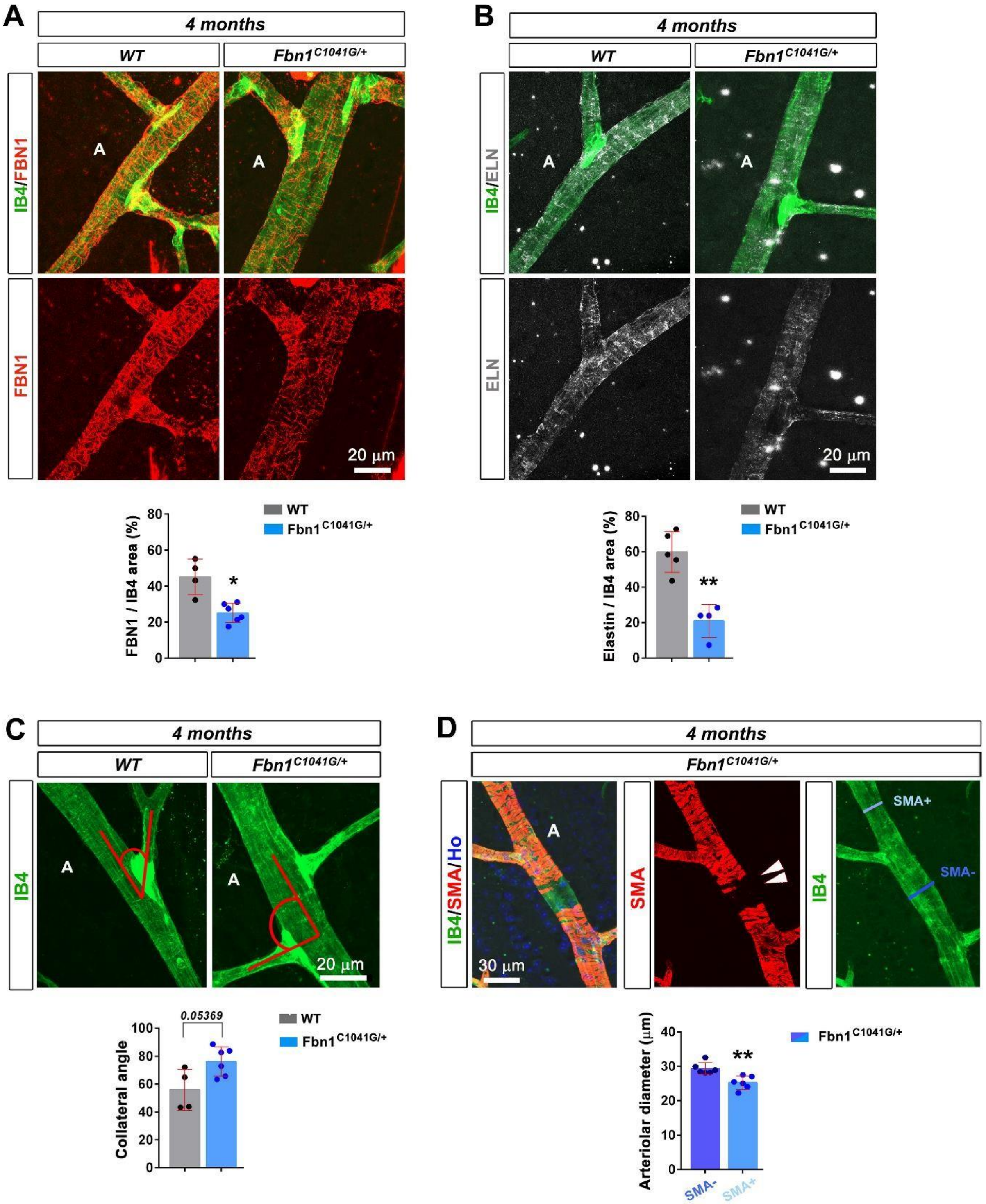


**Figure. S1: Expression of fibrillin-1 and deposition of elastin within the retinal arteriole of adult WT and *Fbn1*$^{C1041G/+}$ mice.**

**A.** IB4 (green) and FBN1 (red) stained retinal arteriole from 4-month-old WT (**left panels**) and *Fbn1*$^{C1041G/+}$ (**right panels**) mice. Quantification of the FBN1 positive area over the IB4 positive area is shown, (n=4 WT and 6 *Fbn1*$^{C1041G/+}$mice). **B.** Same experimental setup as in (**A**) for IB4

(green) and ELN (white), (n=5 WT and 4 *Fbn1*$^{C1041G/+}$mice). **C.** IB4 (green) stained arteriole from *Fbn1*$^{C1041G/+}$ retinas at 4 months (**right panel**) *vs* WT (**left panel**). Quantification of the angle between the main arteriole and its first bifurcation is shown, (n=4 WT and 6 *Fbn1*$^{C1041G/+}$mice). *P<0.05; **P<0.01; *vs* WT mice (Student's t tests). **D.** Retinal arteriole from a 4-month-old *Fbn1*$^{C1041G/+}$ mouse stained for the VSMC marker αSMA (red), IB4 (green) and the nucleus marker Hoechst (blue). Note the focal loss of VSMCs around the retinal arteriole (white arrowheads). Quantification of the arteriolar diameter, measured at areas covered with SMA (light blue line) or devoid of αSMA (dark blue line) are shown. Note that the loss of VSMCs correlates with increased arteriolar diameter (n=6 *Fbn1*$^{C1041G/+}$mice). A; arteriole. **P<0.01 (Student's t tests).

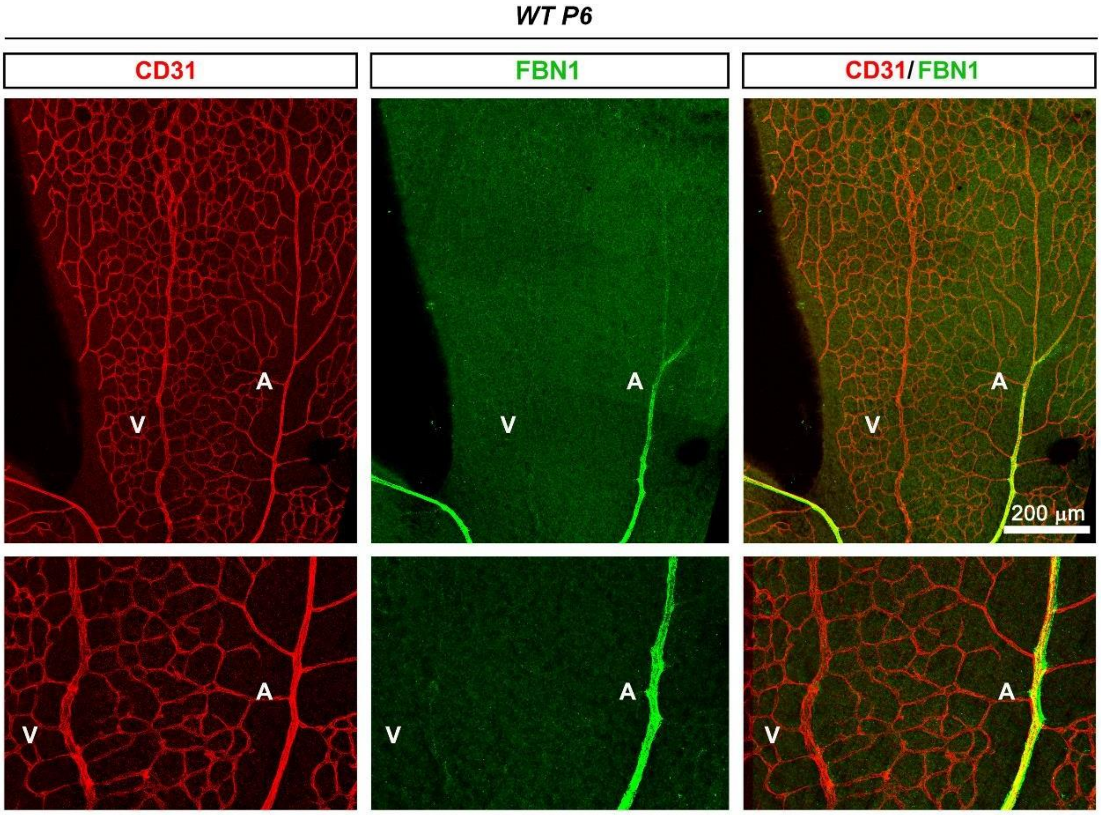


**Figure. S2: FBN1 expression within the microvasculature of the developing mouse retina.** CD31 (red) and FBN1 (green) staining in retina petals from WT mice at P6. At this stage, FBN1 is detected around arterioles but not venules. A; arteriole and V; venule.

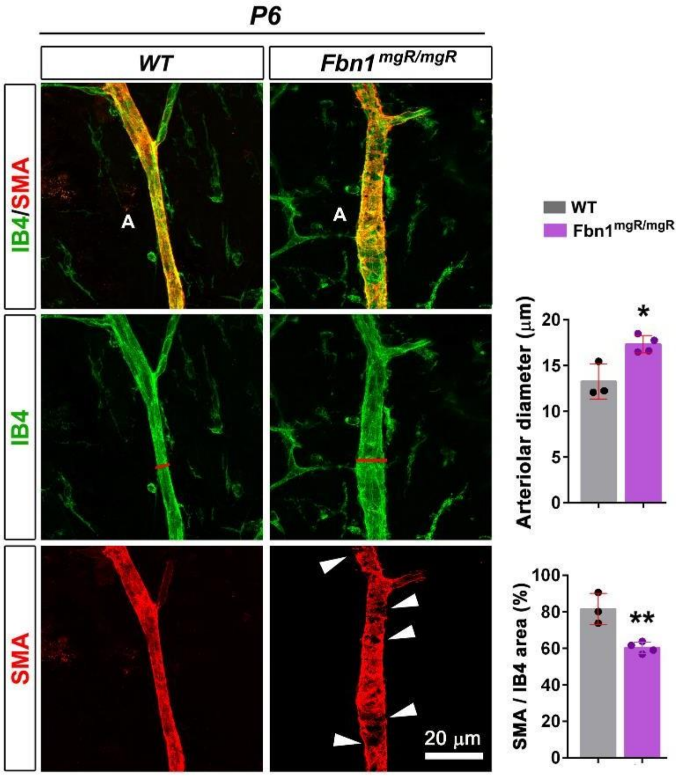


**Figure. S3: Underexpression of fibrillin-1 leads to early alterations in arteriole diameter and αSMA coverage in the developing retina from P6 *Fbn1*$^{mgR/mgR}$ mice**.

Retinal arteriole from 6-day old WT (**left panels**) and *Fbn1*$^{mgR/mgR}$ (**right panels**) mice stained for the VSMC marker αSMA (red) and IB4 (green). Note the increased arteriolar diameter (red line) and the disorganized VSMCs around the retinal arteriole (white arrowheads) in 6-day old *Fbn1*$^{mgR/mgR}$ mice. Quantifications of the arteriolar diameter and the◎SMA positive area over the IB4 positive area are shown, (n=3 WT and 4 *Fbn1*$^{mgR/mgR}$ mice). A; arteriole. *P<0.05; **P<0.01 vs WT mice (Student's t tests).

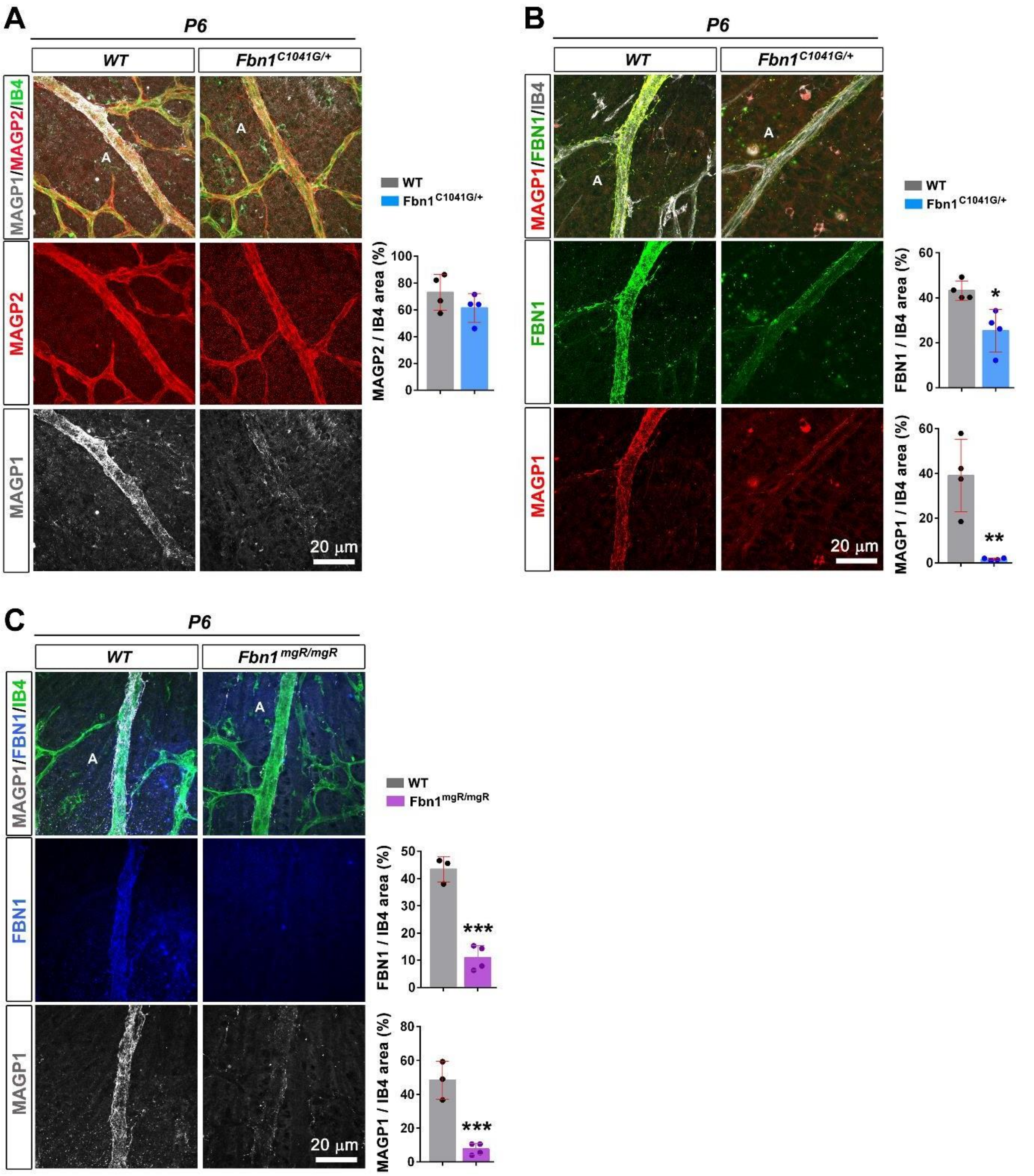


**Figure. S4: Reduced FBN1 and MAGP1 but not MAGP2 expression/deposition in the developing retina from P6 *Fbn1*$^{C1041G/+}$ and *Fbn1*$^{mgR/mgR}$ mice.**

**A.** IB4 (green), MAGP1 (white) and MAGP2 (red) stained retinal arterioles of *Fbn1*$^{C1041G/+}$ retinas at P6 (**right panels**) compared to WT (**left panels**). Quantification of the MAGP2 positive area over the IB4 positive area is shown, (n=4 WT and 4 *Fbn1*$^{C1041G/+}$ mice). A; arteriole. Student's t-test revealed no significant differences between the two groups of mice. **B.** IB4 (white), FBN1 (green) and MAGP1 (red) stained retinal arterioles of *Fbn1*$^{C1041G/+}$ retinas at P6 (**right panels**)

compared to WT (**left panels**). Quantifications of the FBN1 and MAGP1 positive areas over the IB4 positive area are shown, (n=4 WT and 4 *Fbn1*$^{C1041G/+}$ mice). **C.** IB4 (green), FBN1 (blue) and MAGP1 (white) stained retinal arterioles of *Fbn1*$^{mgR/mgR}$ retinas at P6 (**right panels**) compared to WT (**left panels**). Quantifications of the FBN1 and MAGP1 positive areas over the IB4 positive area are shown, (n=3 WT and 4 *Fbn1*$^{mgR/mgR}$ mice). A; arteriole. *P<0.05; **P<0.01; ***P<0.001 vs WT mice (Student's t tests).

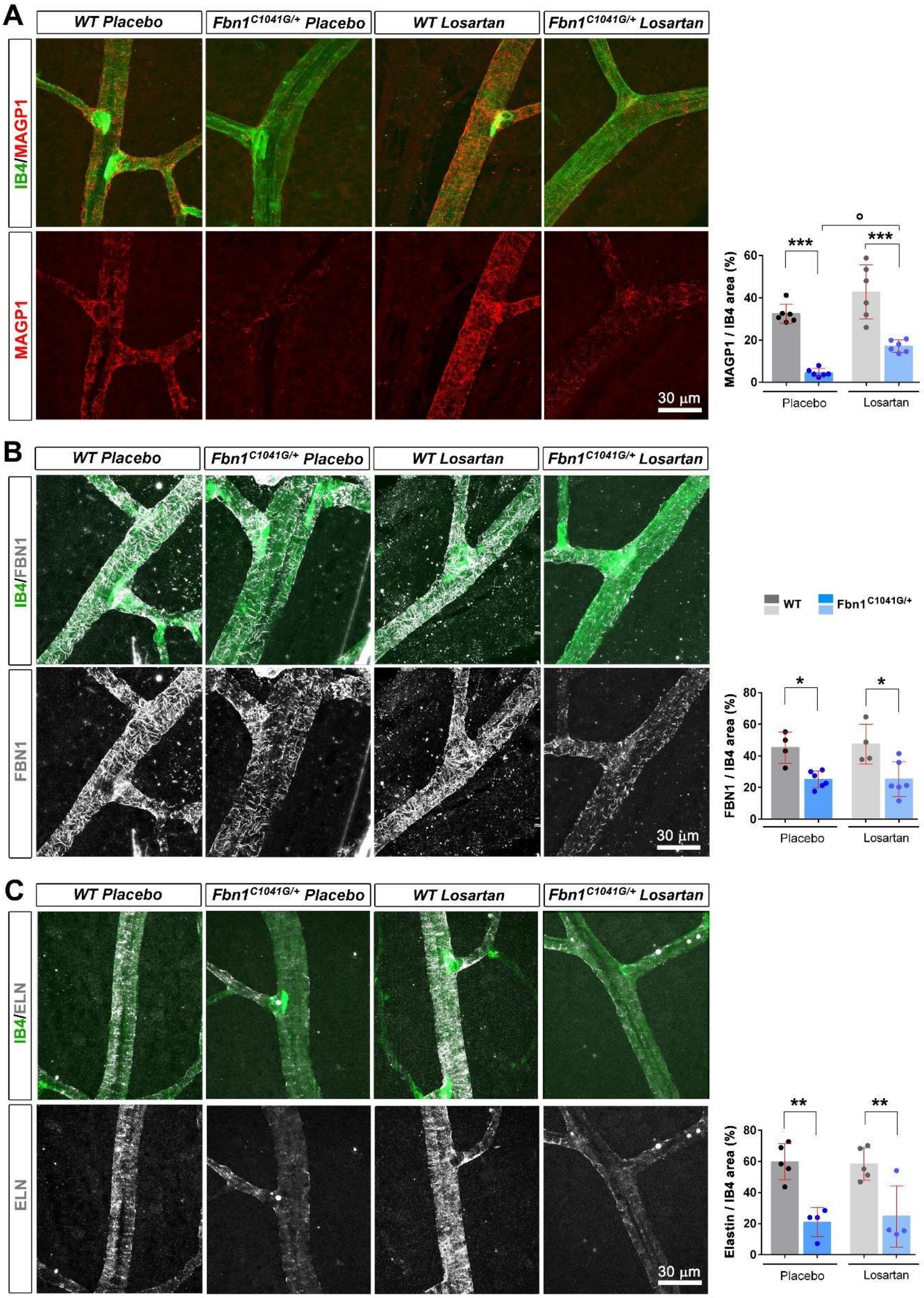


**Figure. S5: Expression/deposition of fibrillin-1, elastin and MAGP1 within the retinal arterioleof adult WT and *Fbn1*$^{C1041G/+}$ mice treated or not with losartan.**

**A.** IB4 (green) and MAGP1 (red) stained retinal arteriole from 4-month-old WT and *Fbn1*$^{C1041G/+}$ mice treated or not with losartan. Quantification of the MAGP1 positive area over the IB4 positive area is shown, (n=6 $H_2O$-treated WT; 6 $H_2O$-treated *Fbn1*$^{C1041G/+}$; 6 losartan-treated WT; 6 losartan-treated *Fbn1*$^{C1041G/+}$ mice). **B-C.** Same experimental setup as in (**A**) for IB4 (green) and FBN1 (white) in (**B)** (n=4 $H_2O$-treated WT; 6 $H_2O$-treated *Fbn1*$^{C1041G/+}$; 4 losartan-treated WT; 6 losartan-treated *Fbn1*$^{C1041G/+}$ mice); for IB4 (green) and ELN (white) in (**C**) (n=5 $H_2O$-treated WT; 4 $H_2O$-treated *Fbn1*$^{C1041G/+}$; 5 losartan-treated WT; 4 losartan-treated *Fbn1*$^{C1041G/+}$ mice). *P<0.05; **P<0.01; ***P<0.001 *vs* respective WT mice. °P<0.05 *vs* respective untreated mice (one-way ANOVA followed by Tukey´s post-tests).

## Supplementary Videos

**Video S1.** 3D volume reconstruction of the area shown in **Fig. 1C** showing that FBN1 is detected as a network around arterioles in close interaction with the endothelial BM in retinas from 4 month old wild type animals (FBN1 (red), IB4 (green), Laminin-γ1 (white) and nucleus (Hoechst, blue)).

**Video S2.** 3D volume reconstruction of the same image of the *Fbn1*$^{C1041G/+}$ arteriole shown in **Fig. 2F** showing focal loss of VSMCs around the retinal arteriole and extravasated albumin in 4 month old *Fbn1*$^{C1041G/+}$ mice (αSMA (red), IB4 (green), albumin (white) and the nucleus marker Hoechst (blue)).

**Video S3**. 3D reconstruction showing that FBN1 is detected around arterioles in wild type retina at P6 (FBN1 (red), IB4 (green) and nucleus (Hoechst, blue))

**Table S1**

| Target antigen | Vendor or Source | Catalog # | Working concentration | Persistent ID / URL |
|---|---|---|---|---|
| Rabbit polyclonal anti-Fibrillin-1 | Reinhardt's laboratory; Tiedemann et al., 2001 | N/A | 1:100 | N/A |
| Rat monoclonal anti-Laminin gamma -1, clone 3E10 | Santa Cruz Biotechnology | Cat# sc-65643; RRID:AB_1123687 | 1:100 | https://www.scbt.com/fr/p/laminin-gamma-1-antibody-3e10 |
| Rabbit polyclonal anti-MAGP-2/MFAP5 | Mybiosource | Cat# MBS2028486 | 1:100 | https://www.mybiosource.com/polyclonal-mfap5-mouse-antibody/microfibrillar-associated-protein-5/2028486 |
| Mouse monoclonal anti-Actin, alpha-Smooth Muscle, Cy3 Conjugated, clone 1A4 | Sigma | Cat# C6198; RRID:AB_476856 | 1:100 | https://www.sigmaaldrich.com/catalog/product/sigma/c6198?lang=fr&region=FR |
| Rabbit polyclonal anti-COLLAGEN IV | Bio-Rad | Cat# 2150-1470; RRID:AB_2082660 | 1:100 | https://www.bio-rad-antibodies.com/polyclonal/mouse-collagen-iv-antibody-2150-1470.html?f=purified |
| Rat monoclonal anti-CD144 (VE-Cadherin), clone 11D4.1 | BD Biosciences | Cat# 555289; RRID:AB_395707 | 1:100 | https://www.bdbiosciences.com/us/applications/research/stem-cell-research/mesoderm-markers/mouse/purified-rat-anti-mouse-cd144-11d41/p/555289 |
| Rat monoclonal anti-CD31 (PECAM), clone MEC13.3 | BD Biosciences | Cat# 553370; RRID:AB_394816 | 1:100 | https://www.bdbiosciences.com/eu/applications/research/stem-cell-research/cancer-research/mouse/purified-rat-anti-mouse-cd31-mec-133/p/553370 |
| Goat polyclonal anti-MAGP-1/MAFP2 | R and D Systems | Cat# AF4977; RRID:AB_2266285 | 1:100 | https://www.rndsystems.com/products/mouse-magp-1-mfap2-antibody_af4977 |
| Goat polyclonal anti-Serum Albumin | R and D Systems | Cat# AF3329 | 1:100 | https://www.rndsystems.com/products/human-mouse-serum-albumin-antibody_af3329 |
| Rabbit polyclonal anti-Tropoelastin | Gift from Dr. Robert P. Mecham | N/A | 1:100 | N/A |
| Alexa Fluor 488 donkey anti-goat IgG antibody | Jackson ImmunoResearch Labs | Cat# 705-546-147; RRID:AB_2340430 | 1:100 | https://www.jacksonimmuno.com/catalog/products/705-546-147 |
| Alexa Fluor 488 donkey anti-rabbit IgG antibody | Jackson ImmunoResearch Labs | Cat# 711-545-152; | 1:100 | https://www.jacksonimmuno.com/catalog/products/711-545-152 |

| | | | | |
|---|---|---|---|---|
| | | RRID:AB_2313584 | | |
| Alexa Fluor 488 donkey anti-rat IgG antibody | Jackson ImmunoResearch Labs | Cat# 712-545-153 RRID:AB_2340684 | 1:100 | https://www.jacksonimmuno.com/catalog/products/712-545-153 |
| Alexa Fluor 647 donkey anti-goat IgG antibody | Jackson ImmunoResearch Labs | Cat# 705-605-003; RRID:AB_2340436 | 1:100 | https://www.jacksonimmuno.com/catalog/products/705-605-003 |
| Alexa Fluor 647 donkey anti-rabbit IgG antibody | Jackson ImmunoResearch Labs | Cat# 711-606-152; RRID:AB_2340625 | 1:100 | https://www.jacksonimmuno.com/catalog/products/711-606-152 |
| Alexa Fluor 647 donkey anti-rat IgG antibody | Jackson ImmunoResearch Labs | Cat# 712-605-153; RRID:AB_2340694 | 1:100 | https://www.jacksonimmuno.com/catalog/products/712-605-153 |
| Cy™3 donkey anti-goat IgG antibody | Jackson ImmunoResearch Labs | Cat# 705-166-147; RRID:AB_2340413 | 1:100 | https://www.jacksonimmuno.com/catalog/products/705-166-147 |
| Cy™3 donkey anti-rabbit IgG antibody | Jackson ImmunoResearch Labs | Cat# 711-165-152; RRID:AB_2307443 | 1:100 | https://www.jacksonimmuno.com/catalog/products/711-165-152 |
| Cy™3 donkey anti-rat IgG antibody | Jackson ImmunoResearch Labs | Cat# 712-165-153; RRID:AB_2340667 | 1:100 | https://www.jacksonimmuno.com/catalog/products/712-165-153 |
